# Quantitative SWATH-Based Proteomic Profiling for Identification of Mechanism-Driven Diagnostic Biomarkers Conferring in the Progression of Metastatic Prostate Cancer

**DOI:** 10.3389/fonc.2020.00493

**Published:** 2020-04-08

**Authors:** Anshika N. Singh, Neeti Sharma

**Affiliations:** ^1^Symbiosis School of Biological Sciences, Symbiosis International (Deemed University), Pune, India; ^2^School of Engineering, Ajeenkya DY Patil University (ADYPU), Pune, India

**Keywords:** SWATH, prostate cancer, proteomics, biomarkers, EMT

## Abstract

Prostate cancer (PCa), the most frequently diagnosed malignancy in men is associated with significant mortality and morbidity. Therefore, demand exists for the identification of potential biomarkers for patient stratification according to prognostic risks and the mechanisms involved in cancer development and progression to avoid over/under treatment of patients and prevent relapse. Quantitative proteomic mass spectrometry profiling and gene enrichment analysis of TGF-β induced-EMT in human Prostate androgen-dependent (LNCaP) and androgen-independent (PC-3) adenocarcinoma cell lines was performed to investigate proteomics involved in Prostate carcinogenesis and their effect onto the survival of PCa patients. Amongst 1,795 proteins, which were analyzed, 474 proteins were significantly deregulated. These proteins contributed to apoptosis, gluconeogenesis, transcriptional regulation, RNA splicing, cell cycle, and MAPK cascade and hence indicating the crucial roles of these proteins in PCa initiation and progression. We have identified a panel of six proteins viz., GOT1, HNRNPA2B1, MAPK1, PAK2, UBE2N, and YWHAB, which contribute to cancer development, and the transition of PCa from androgen dependent to independent stages. The prognostic values of identified proteins were evaluated using UALCAN, GEPIA, and HPA datasets. The results demonstrate the utility of SWATH-LC-MS/MS for understanding the proteomics involved in EMT transition of PCa and identification of clinically relevant proteomic biomarkers.

## Introduction

Prostate cancer (PCa) in men is known to be the most frequently diagnosed noncutaneous solid organ cancer and the second major cause of cancer mortalities in the United States (1). In the current scenario, the prostate tumor is observed as a growing indolent tumor or advanced aggressive cancer however according to evidence the currently available diagnostic biomarker prostate-specific antigen (PSA) and histological examinations of tumor tissues cannot completely specify the tumor stages, as well as its aggressiveness (2). Reports have shown that almost 90% men with PCa have localized tumors that may lead to under/over-treatment, which may lead to mortality. So far, the available treatments include radiation therapy, hormonal therapy, and surgery that carry certain risks of complications and have known to escort side effects, which may hamper the patient's long term quality of life (2, 3). Hence, there exists an urgent need to identify new clinical biomarkers for PCa that may distinguish different PCa stages. In clinical setup, the histological samples of tumor biopsies are graded on the basis of Gleason score (2–10) that indicates the aggressiveness of the tumor and its metastatic potential (3). Unfortunately, Gleason score can fail at times due to molecular and clinical heterogeneities of Prostate tumors.

Tumor cells are known to undergo dynamic changes for acquisition of invasive properties and epithelial to mesenchymal transition (EMT) plays a vital role in epithelial tumor cell metastasis. EMT is a developmental process which is characterized by downregulation of epithelial features and upregulation of the mesenchymal phenotype (4). Among these characteristics, loss of E-cadherin which is an epithelial marker is often associated with progression of PCa and Gleason score grading, hence pointing toward EMT playing crucial role during PCa metastasis. EMT is also known to be involved with tissue formation and also organ development. Transforming growth factor (TGF-β) is a known EMT inducer in epithelial cells and in most cases mandatory for acquisition of invasive properties in carcinoma cells. (4)

TGF-β, a multifunctional cytokine is a key regulator in tumorigenesis viz., proliferation, differentiation, and apoptosis. TGF-β is known to play a paradoxical role in cancer biology wherein it functions both as a tumor promoter and tumor suppressor (5). Numerous reports have suggested increased expression of TGF-β directly affecting enhanced invasion and metastasis of neoplastic cells. Evidence shows, EMT promotes PCa progression and is closely related to increased stemness and therapeutic resistance (5, 6). EMT phenotype is characterized by loss of E-cadherin and expression of mesenchymal proteins, including N-cadherin, vimentin, and fibronectin. Transcriptional repression of E-cadherin and induction of mesenchymal phenotype can be facilitated by TGF-β in cancer cells (4–6). Several reports have suggested that TGF-β1 can induce EMT in prostate epithelial cells and also in mouse tumor model by targeting deletion of SMAD3, and hence indicating toward potential of TGF-β signaling in prostatic cancer metastasis. TGF-β can also induce EMT through active Akt which consequently inhibits SMAD3 and p21 translocation to the nucleus. PCa cells tend to undergo EMTs via interactions between TGF-β1 and Androgen receptors. Numerous reports have established that these interactions between TGF-β and Androgen signaling play a determining role in prostate tumor growth, invasion and metastasis by regulating apoptosis, EMT and also via remodeling of actin cytoskeleton (6). In addition to that, a crosstalk between TGF-β and androgen axis potentially contributes to the functional switch of TGF-β from a tumor suppressor to a prompter of tumor metastasis in preclinical models of PCa progression at both *vitro* and in *in vivo* levels (6).

In this study, we have performed SWATH-LC-MS/MS analysis for quantitative comparisons of proteomics involved in the transition of PCa from androgen dependent to androgen independent stage by induction of exogenous TGF-β and further have reviewed the effect of identified proteins on the long-term survival of patients.

So far, researchers worldwide are using transcriptome profiles due to the advancement and availability of different measurement techniques (7). But current research has shown the emergence of proteomic measurements as excellent biomarkers since proteins are considered to be more diverse, dynamic, and reflective of cellular physiology as compared to genomic markers. Also, currently available diagnostic protein markers such as PSA vouch for the potential of protein markers (8).

The proteome analysis of human samples using mass spectrometric based techniques is being thoroughly considered for analysis of cancer biomarkers. Another emerging proteomic analysis technique viz., SWATH (The Sequential Window Acquisition of All Theoretical Fragment Ion Mass Spectra) on combination with LC-MS/MS can be used for searching relevant ion datasets by merging data-independent acquisition which is highly specific with novel data extraction strategies (9). The major advantage of SWATH is that being a label-free analysis, it can be easily combined with liquid chromatography-mass spectrometry (LC-MS/MS), which is both conventional and comprehensive, and reliable quantification of potential protein markers can be achieved by SWATH-LC-MS/MS screening by setting up strict filtration criteria and further enrichment analysis (10, 11).

## Materials and Methods

### Cell Culture

Two PCa cell lines viz., androgen-dependent LNCaP cell line, and androgen-independent PC-3 cell line were selected for the study, which broadly represents the clinical scenario of PCa stages. The LNCaP cell line is androgen-sensitive, less malignant, less metastatic, and possess epithelial features. PC-3 cells are androgen independent, malignant, metastatic, and possess mesenchymal features when compared to other PCa cell lines such as LNCaP, VCaP, and RWPE1. The PC-3 and LNCaP human prostate adenocarcinoma cell lines were obtained from National Centre for Cell Science (NCCS), Pune, and maintained in RPMI-1640 supplemented with 10% fetal bovine serum, penicillin, and streptomycin (Himedia Laboratories Pvt. Ltd., India). Both the cell lines were then subjected to doses of human recombinant TGF-β (Himedia Laboratories Pvt. Ltd., India) for inducing EMT. The treatment dosages of TGF-β were calculated post-MTT assay for IC50 evaluations. All the experiments were performed in triplicates.

### Quantitative Real Time PCR and Western Blot

The RNA was then extracted from LNCaP and PC-3 cells in cells in naïve state and post-TGF-β treatment using TRI reagent (Sigma). The total RNA was then estimated using Thermo NanoDrop UV spectrophotometer. The cDNA was prepared from the total RNA by High-capacity cDNA Reverse Transcription kit. Real time polymerase chain reaction (PCR) was carried out using SYBR green dye and the endogenous control used in the analysis was β-actin, and the expressions were analyzed using comparative CT method. The primers used in our analysis were: E-cadherin (Epithelial marker), N-cadherin, and Vimentin (Mesenchymal markers).

For Western blot analysis, PC-3 cells and LNCaP cells were harvested after 24 h of TGF-β treatment. 1X RIPA buffer with 1X protease inhibitor cocktail was used to lyse the cells. The protein samples were estimated by Bradford assay. The equivalent amounts of protein samples (30 μg) were separated using 10% SDS-PAGE gels and transferred to nitrocellulose membrane. The membrane was then incubated with primary antibodies against E-cadherin (Santa Cruz Biotechnologies, Cat.#sc-8426, 1:200 dilution) and N-cadherin (Santa Cruz Biotechnologies, Cat.#sc-271386, 1:200 dilution) overnight at 4°C. β-actin (BD Biosciences, Cat.#612656, 1:1,000 dilution) was used as an endogenous control. The membrane was then incubated with secondary anti-mouse IgG antibody (Sigma Aldrich, Cat.#A9077, 1:10,000 dilution) for an hour at room temperature. The blots were visualized using Lumiglo on a Syngene G: Box imaging system.

### Protein Extraction and In-solution Digestion

For protein extraction, after homogenizing the samples in 1 mL of lysis buffer (1 × protease inhibitor cocktail) the samples were lysed by sonication on ice. Post-sonication, the supernatant was collected by centrifuging the samples at 17,000 rpm for 1 h at 4°C, and then used for protein concentration determination by the Bradford method at an absorbance of 595 nm. We then proceeded for In-solution digestion using ammonium carbonate, Dithiothreitol (DTT), activated trypsin solution, and 20 mM Iodoacetamide (IAA).

### Desalting and Sample Preparation

Three solutions of 100% Acetonitrile, 50% Acetonitrile + 0.1% Formic Acid, and 0.1% Formic Acid in LCMS grade water were prepared. The samples were then equilibrated in the three solutions, and then 20 μg proteins were bound aspirated and then dispensed in the peptide solution for 5–10 times followed by speed vacuuming at 25°C. The protein samples were then prepared in 3% ACN with 0.1% Formic Acid. For SWATH analysis, the maximum sample load was taken as 2 μg, and B-gal standard digest spiking was considered at 200 fm.

### SWATH-LC-MS/MS Analysis

For the mass spectrometry analysis, AB Sciex T.TOF 5,600 mass spectrometer coupled to Eskigent MicroLC 200 system (Eskigent, Dublin CA) equipped to Eskigent C18-reverse-phase column (100 ×0.3 mm, 3 μm, 120 Å) was employed for data extraction and spectral alignment of protein samples. Digested peptides were then injected into the column followed by eluting the peptides at 90 mm linear gradient of 3–50% ACN. The acquisition and processing for the samples was carried out using Analyst TF Software 1.6. High-quality spectral ion libraries were generated by subjecting samples to data-dependent analysis strategy and the proteins were identified via Protein Pilot 5.0 software (AB Sciex) using Paragon Algorithm against the Human Proteome database (1% globar FDR). This was followed by reviewing the protein molecule markers using PeakView 2.2 and normalizing the protein intensity peak areas in MarkerView (Version 1.2.1, AB Sciex). In PeakView 2.2, the processing parameters were sets as Number of peptides per protein: 6, number of transitions per peptide: 10; peptide confidence threshold: 6 and FDR 1% XIC extraction window; 5 min and width 50 ppm. The data was then subjected to Log_2_ transformation before statistical analysis. Histograms were plotted to evaluate the normality distribution of individual protein samples. Fold change was calculated for each of the protein expression changes observed during the run. Based on previous literature, we set the threshold expression fold change (FC) as FC ≥ 1.5 or FC ≤ 1.5 with adjusted *p* ≤ 0.05 for proteins to be considered statistically significant for all the differentially expressed proteins (DEPs).

### Gene Ontology and Functional Enrichment

For carrying out pre-processing of the proteins for gene ontology, each of the Protein IDs was converted to their Generic Gene Names using UniProt (12). Gene Ontology and functional enrichment were performed using PantherDB (13) and GeneCodis (14). The Gene Ontology results were categorized into three categories namely Biological process (BP), Molecular Functions (MF), and Cellular compartment localization (CC). The results with a *p*-value of 0.01 were considered to be significant. The enriched pathways were analyzed using Reactome (15) and Kyoto Encyclopedia of Genes and Genomes (KEGG) (16). The interrelationship of identified proteins in regulating cellular pathways was retrieved using NetworkAnalyst (17).

### PPI Network Construction

The Search Tool for the Retrieval of Interacting Genes (STRING) was employed for constructing the protein-protein interaction network for the identification of hub genes (18). Experimentally validated Interactions with ≥ 0.7 (high confidence) were considered to be significant during this analysis.

### Correlation Between mRNA and Protein Expression and Clinicopathological Parameters of PCa Patients

The mRNA expression levels of identified six proteins were correlated with The Cancer Genome Atlas (TCGA) PCa datasets and GTEx datasets using the online GEPIA (Gene Expression Profiling Interactive Analysis) database (19). The protein expression levels of these identified proteins in PCa tumor samples were then analyzed using The Human Protein Atlas Database (20).

### Survival Analysis

Survival analysis was studied using the UALCAN website, which is a portal based on TCGA dataset (21). The PCa samples were categorized into high and low/median expression based on their Transcripts per million [TPM]. The prognostic values of the proteins were then analyzed on the basis of the Kaplan-Meier method (*p* < 0.05).

## Results

### Prostate Cancer Cell Lines and Treatment

The cell lines were maintained according to the ATCC guidelines and LNCaP cells were harvested between passages 30 and 35 which are considered to be low passage numbers and PC3 cells were used between passages 17 and 21. The cell lines were then subjected to TGF-β (10 ng/ml) treatment to induce EMT post performing cell viability assays for 24 h in both cell lines (Figure S1). To ensure successful induction of EMT post-TGF-β treatment, we further verified its effects on expression levels of epithelial marker (E-cadherin) and mesenchymal markers (N-cadherin and Vimentin) at both gene and protein levels (Figure S2).

The expression levels of the characteristic markers of EMT viz., E-cadherin, N-cadherin, and Vimentin were evaluated by real-time PCR and Western blot to determine the effectiveness of the TGF-β treatment on EMT markers. The analysis showed that the treatment with TGF-β downregulated the expressions of E-cadherin with significant upregulation of mesenchymal markers (N-cadherin and Vimentin) in both androgen-dependent and androgen-independent PCa cell lines.

### Differentially Expressed Proteins Quantified by SWATH-LC-MS/MS Analysis in Prostate Cancer Cell Lines

**Figure 1:**
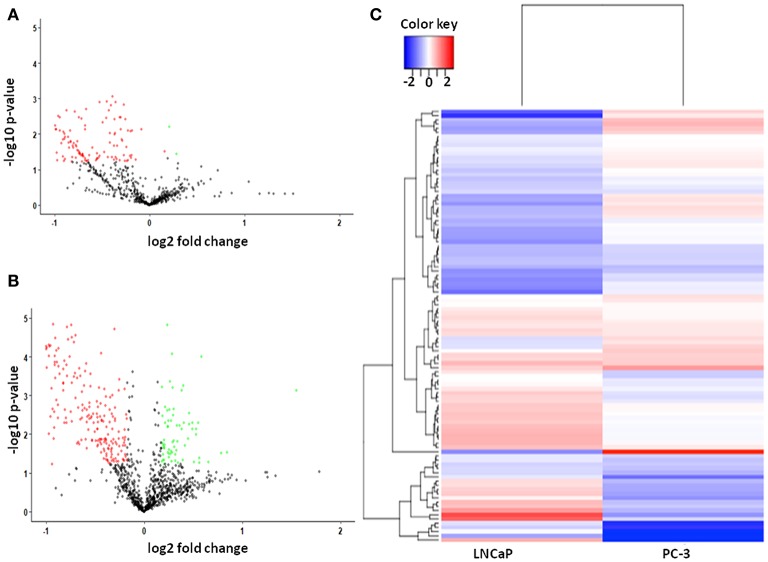
**(A)** Scatter plot of 660 dysregulated proteins, including 2 upregulated and 126 downregulated proteins in Androgen dependent Prostate cancer cell lines. The green dots are representatives of upregulated proteins and red dots represent the downregulated proteins; **(B)** Scatter plot of 1,135 dysregulated proteins, including 69 upregulated and 277 downregulated proteins in Androgen independent Prostate cancer cell lines. The green dots are representatives of upregulated proteins and red dots represent the downregulated proteins. **(C)** Heatmap of dysregulated proteins in both Androgen dependent and Androgen independent cell line. The color bar represents the expression levels of the proteins, corresponding to the log_2_-ratio of fold change.

The protein expression phenotypes of PCa cell lines were investigated by SWATH data-independent quantitative mass spectrometry to compare DEPs during both androgen-dependent and independent stages in LNCaP and PC-3 by inducing EMT using TGF-β. In total, post the SWATH-LC-MS/MS analysis, we identified in total 1795 proteins to be differentially regulated in samples comprising of androgen dependent and androgen independent PCa cells, among which 474 proteins were observed to be significantly regulated using the MarkerView software run against the Human Proteome Database. In LNCaP, among 660 differentially regulated proteins, 2 proteins were observed to be significantly upregulated, and 126 proteins were seen to be downregulated (Figure 1A). In the androgen-independent PC-3 cell line, we observed 1,135 proteins in total to be regulated, among which 69 proteins were significantly upregulated, and 277 proteins were observed to be significantly downregulated (Figure 1B). The analysis identified 26 proteins to be significantly regulated in both androgen-dependent (LNCaP) and androgen independent (PC-3) cell lines (Figure 1C). Hence, these 26 proteins are not affected by androgen signaling which is known to be a key player in transition of PCa from androgen dependent to androgen independent stage and hence contributes in both stages of PCa.

### Gene Ontology

**Figure 2:**
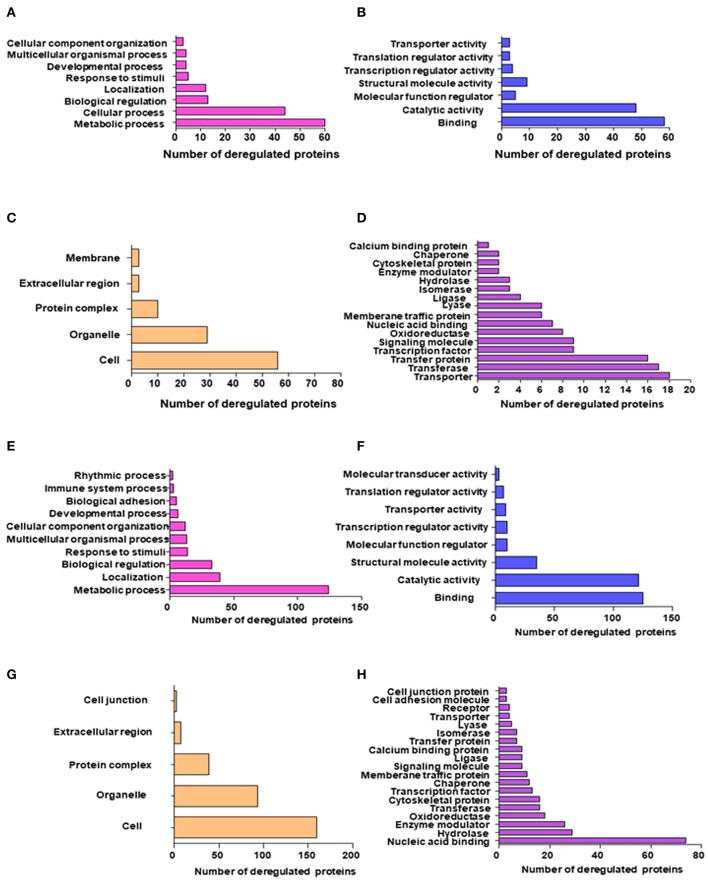
GO and KEGG pathway enrichment of most significantly regulated proteins in Androgen dependent cell lines post-TGF-β treatment; **(A)** Top significantly enriched biological processes of deregulated proteins quantified using the SWATH-LC-MS/MS approach; **(B)** Top significantly enriched protein Molecular processes of deregulated proteins quantified using the SWATH-MS approach; **(C)** Top significantly enriched Cellular localization of deregulated proteins quantified using the SWATH-LC-MS/MS approach; **(D)** Top significantly enriched protein class of deregulated proteins quantified using the SWATH-LC-MS/MS approach; GO and KEGG pathway enrichment of most significantly regulated proteins in Androgen independent cell lines post-TGF-β treatment; **(E)** Top significantly enriched biological processes of deregulated proteins quantified using the SWATH-LC-MS/MS approach; **(F)** Top significantly enriched protein Molecular processes of deregulated proteins quantified using the SWATH-LC-MS/MS approach; **(G)** Top significantly enriched Cellular localization of deregulated proteins quantified using the SWATH-MS approach; and **(H)** Top significantly enriched protein class of deregulated proteins quantified using the SWATH-LC-MS/MS approach.

The altered biological function and MF of the identified DEPs were then analyzed using PANTHER and GENECODIS (Figure 2). In the BP—Gene ontology, the most significantly regulated BP included metabolic process, biological adhesion, rhythmic process, immune system response, and response to stimuli. During the Molecular Function—Gene ontology analysis, we found that the majority of DEPs contributed specifically to Nucleotide and protein binding, Transporter activity, Transcription regulator activity, and catalytic activity. Other regulated MF includes oxidoreductase activity, SMAD binding, and protein phosphate binding. Other regulated biological processes included the catabolic process and their regulation, generation of metabolites and energy, translation, RNA splicing, Hypoxia, DNA damage response, and various cell cycle stages. The DEPs also regulated response to hypoxia, DNA damage regulation of cell shape and signal transduction. The DEPs were seen to be mostly localized in the cell organelles, extracellular region, and cell junction. The proteins identified by SWATH-LC-MS/MS strategy belonged to nucleic acid binding, Hydrolase, Enzyme modulator, Membrane traffic proteins, and signaling molecules proteins classes. The most significantly enriched pathways identified using KEGG analysis include RNA transport, TCA cycle, Glycolysis, Pentose phosphate pathway, Cell cycle, and Pyruvate metabolism (Figure S3). The other biological processes include cellular localization and its establishment, cofactor metabolic process, heterocyclic metabolic process, and response to stimuli and chemicals. KEGG pathway analysis showed DEPs contributed in Glycolysis, Protein processing in the endoplasmic reticulum, Pathways in cancer, Regulation of actin cytoskeleton, and vasopressin-regulated water reabsorption. Panther pathway analysis showed the contribution of DEPs in the apoptosis signaling pathway, cadherin signaling, integrin signaling, and ubiquitin-proteasome pathway, angiogenesis, which are known to play a significant role in tumorigenesis (Figure S4). Based on our analysis, proteins that contributed most significantly in the initiation and progression of androgen-dependent PCa in an induced-EMT stage include HADHA, GAPDH, ALDOC, MDH2, GOT1, and HSP90B1 (Figure 1A). The most significantly differentially regulated proteins seen post-TGF-β induced EMT in androgen-independent PC-3 cell line included proteins., ALDOC, PURA, SEC24B, PDHA1, CLH1, CLPP, AK1A1, PURB, PTGR1, SEC22B, AIMP1, BCL2, and SEC13 (Figure 1B). Based on common protein analysis, we found that there were 26 proteins that were differentially regulated significantly in both androgen dependent and androgen independent stages of cancer viz., TERA, TKT, GAPDH, HNRPD, HSPA4, PURB, GOT1, PSMA7, CS, UBA3, ALDOC, GNS, ECHB, ALB, 6PGD, HNRNPH3, STRAP, SPTAN1, FAH, LA, YWHAB, TAGLN2, HNRNPA2B1, LASP1, KTN1, and FKBP4 (Figure 1C). These proteins were seen to be significantly contributing to carbohydrate metabolic process, apoptotic process, gluconeogenesis, negative regulation of transcription, RNA splicing, cellular nitrogen compound metabolic process and TGF-β-beta receptor signaling and hence implying toward the crucial roles of these identified proteins in PCa initiation and progression. Thus, these identified proteins also contribute significantly to the transition of androgen-dependent PCa to androgen-independent PCa stage.

### PPI Network

For identification of hub genes, we categorized the identified differentially regulated proteins based on thorough literature survey and gene ontology results depicting their contribution in cancer hallmarks and shortlisted 14 proteins for further evaluation of their potential as biomarkers by studying their interactions and influence on survival of PCa patients (Figure S5). The protein-protein interaction network showed among analyzed 14 proteins with 37 edges, six proteins emerged to be considered as hub genes for further analysis. These proteins showed maximum interactions with other analyzed proteins and hence could be considered to be considered playing a crucial role during onset and progression of PCa (Table 1).

**Table 1:** Differentially expressed proteins in androgen dependent and androgen independent prostate cancer cell lines identified using SWATH-LC-MS/MS.

**S.No**	**Protein** **(Human)**	**UNIProt ID**	**Protein Name**	**Occurrence**	**Contribution**
1	GOT1	P17174	Aspartate aminotransferase	LNCaP PC-3	GO-MF: carboxylic acid binding; GO-MF: phophatidyl decarboxylase activity; GO-MF: pyridoxal phosphate binding; GO-BP: aspartate metabolic process; GO-BP: gluconeogenesis; GO-BP: notch signaling; GO-BP: transdifferentiation
2	HNRNPA2B1	P22626	Heterogeneous nuclear ribonucleoproteins A2/B1	LNCaP	GO-MF: protein binding; GO-MF: mRNA binding; GO-MF: N6-methyladenosine containing RNA binding; GO-BP: miRNA transport; GO-BP: mRNA processing; GO-BP: RNA transport; GO-BP: telomere maintenance
3	MAPK1	P28482	Mitogen-activated protein kinase 1	PC-3	GO-MF: ATP binding; GO-MF: kinase activity; GO-MF: phosphatase binding; GO-MF: transcription factor binding; GO-BP: activation of MAPK activity; GO-BP: cell cycle; GO-BP: cellular response to DNA damage; GO-BP: response to tumor necrosis; GO-BP: response to reactive oxygen species
4	PAK2	Q13177	Serine/threonine-protein kinase PAK2	PC-3	GO-MF: ATP binding; GO-MF: cadherin binding; GO-MF: protein kinase binding; GO-MF: protein tyrosine kinase activator activity; GO-BP: apoptotic process; GO-BP: cell migration; GO-BP: protein phosphorylation; GO-BP: MAPK cascade; GO-BP: T cell receptor signaling
5	UBE2N	P61088	Ubiquitin-conjugating enzyme E2 N	LNCaP PC-3	GO-MF: ATP binding; GO-MF: ubiquitin protein ligase binding; GO-MF: ubiquitin-protein transferase activity; GO-BP: MAPK activity; GO-BP: protein modification; GO-BP: DNA and post replication repair; GO-BP: T cell receptor signaling
6	YWHAB	P31946	14-3-3 protein beta/alpha	LNCaP; PC-3	GO-MF: cadherin binding, GO-MF: histone deacetylase binding; GO-MF: protein-domain specific binding; GO-BP: hippo signaling; GO-BP: MAPK cascade; GO-BP: regulation of G protein-coupled receptor; GO-BP: transcriptional regulation; GO-BP: protein targeting

### Validation of the Identified Hub Proteins

**Figure 3:**
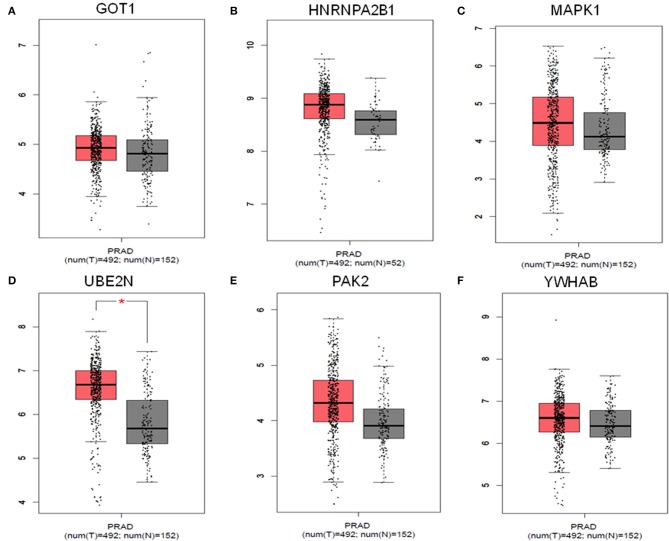
Comparisons of the expression of the six genes between Prostate cancer (*T* = 492) and non-Prostate cancer tissues (*N* = 152) in TCGA and GTEx based on GEPIA. The *Y*-axis represents the log_2_(TPM + 1) for gene expression. The gray bar represents the normal (*N* = 152) non-PCa tissues, and the red bar shows the Prostate cancer (*T* = 492) tissues. TPM, transcripts per kilobase million; *p* < 0.05. **(A)** GOT1, **(B)** HNRNPA2B1, **(C)** MAPK1, **(D)** UBE2N, **(E)** PAK2, and **(F)** YWHAB.

**Figure 4:**
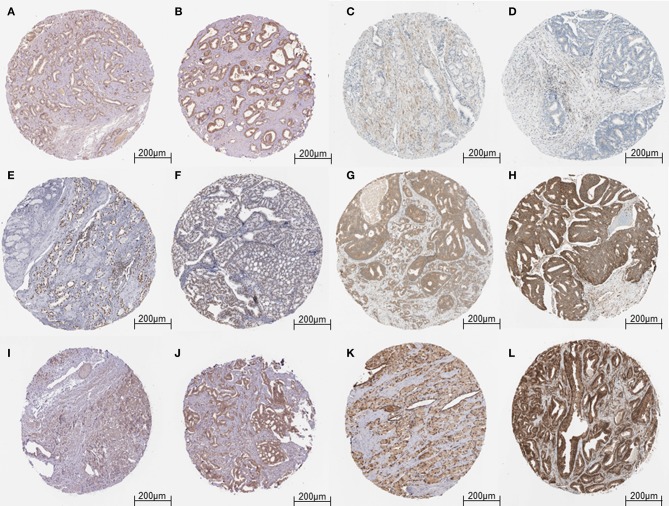
Validation of the hub genes from the HPA database. **(A)** Representative image of Low expression of GOT1 in Prostate tumor cells. **(B)** Representative image of Medium expression of GOT1 in Prostate tumor cells. **(C,D)** Representative image of Low expression of PAK2 in Prostate tumor cells. **(E,F)** Representative image of High expression of HNRNPA2B1 in Prostate tumor cells. **(G)** Representative image of Medium expression of MAPK1 in Prostate tumor cells. **(H)** Representative image of High expression of MAPK1 in Prostate tumor cells. **(I)** Representative image of Low expression of UBE2N in Prostate tumor cells. **(J)** Representative image of Medium expression of UBE2N in Prostate tumor cells. **(K)** Representative image of Medium expression of YWHAB in Prostate tumor cells. **(L)** Representative image of High expression of YWHAB in Prostate tumor cells.

The results obtained from GEPIA analysis showed that all the identified six hub genes were seen to be significantly upregulated in Prostate tumor samples (*n* = 492) when compared to normal prostate samples (*n* = 152; Figure 3). Thus, using GEPIA, the selected six hub proteins were further verified as differentially expressed in PCa with amplified normal sample sizes. The immunohistochemical analysis of the shortlisted proteins viz., GOT1 (Figures 4A,B), PAK2 (Figures 4C,D), HNRNPA2B1 (Figures 4E,F), MAPK1 (Figures 4G,H), UBE2N (Figures 4I,J), and YWHAB (Figures 4K,L), retrieved from the Human Protein Atlas also showed higher expression patterns of these proteins in PCa tissues when compared to normal samples.

### Survival Analysis of Identified Differentially Expressed Proteins

**Figure 5:**
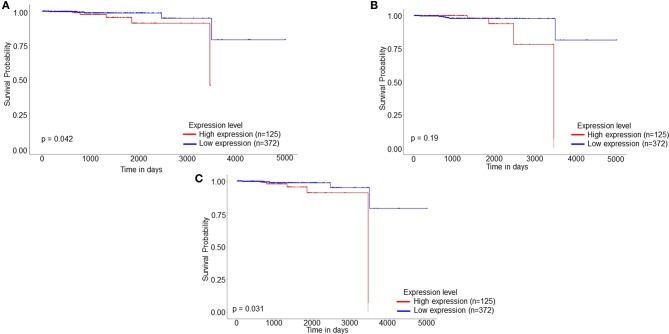
Kaplan Meier patient survival plot based on SWATH-LC-MS/MS protein area of **(A)** HNRNPA2B1, **(B)** UBE2N, and **(C)** PAK2. The red line represents High expression (*n* = 125) and the blue line represents Low/Medium expression (*n* = 372).

PCa patients' data was downloaded from the TCGA database and subjected to Kaplan Meier survival analysis. The test was applied to evaluate the influence of expression status of identified proteins on the long-term survival of PCa patients. Online analysis database UALCAN was employed for analyzing the gene expression data and the related long term patient survival information based on the available cancer transcriptome data. Based on thorough survival analysis of TCGA expression data, the shortlisted differentially regulated six proteins were submitted to UALCAN website for data analysis by employing the Kaplan Meier log-rank test strategy. The survival analysis showed that the patients with high expressions of GOT1, HNRNPA2B1, MAPK1, PAK2, UBE2N, and YWHAB showed shorter overall survival of PCa patients when compared to the low/intermediate expression of these proteins. The survival analysis further revealed that the high expression of identified proteins: PAK2, HNRNPA2B1, and ALDOC significantly affected the overall long-term survival of PCa patients (Figure 5).

## Discussion

The focus of this study was to understand the proteomic variation in PCa progressive stages viz., androgen-dependent to androgen-independent stages and also during an induced EMT stage. This aims to provide key proteins as potential candidates for biomarker discovery and also shed light on the molecular mechanisms involved in PCa progression to metastatic stages. Although a lot of research has already been carried out to understand the mechanism of PCa, there still exists a lacuna in understanding the biological mechanisms involved in the transition of PCa from androgen dependent to androgen-independent stages.

In contrast to previous literature that tumor metastasizes in a linear and stepwise manner, recent reports demonstrate that a section of tumors has molecular alterations at an early stage itself which consequently leads to them reaching metastasis and resulting in bad prognosis and poor survival rate (22). The current tools for detection of PCa which is PSA, etc., fail to detect such molecular changes (23). Hence, there is an urgent need for developing prognostic markers that may predict recurrence and identifying high-risk patients at an early stage.

According to our study, based on a thorough literature survey and online databases, Gene ontology descriptions depicted the involvement of these proteins in cell motility, adhesion, cytoskeleton architecture, cell cycle, and apoptosis. Here, we hypothesized that these identified DEPs could potentially provide more insights in the proteomic programming involved in PCa progression during TGF-β induced metastasis. The gene ontology results further supported our hypothesis, by depicting the contribution of DEPs in regulating several mechanisms related to hallmarks of cancer including apoptosis signaling pathway, biological adhesion, immune system response, response to stimuli, cadherin signaling, developmental response, ubiquitin-proteasome pathway, and angiogenesis.

Finally, using a protein-protein interaction network, six proteins viz., GOT1, HNRNPA2B1, MAPK1, PAK2, UBE2N, and YWHAB were detected as hub proteins. The differential mRNA and protein expressions of these identified using GEPIA and HPA further revealed these proteins to be upregulated in PCa tissue samples when compared to normal prostate tissue samples.

Our analysis showed that UBE2N to be significantly dysregulated in both LNCaP and PC-3 cell lines and also affecting the long-term survival of patients (Figure 5). The ubiquitin conjugation enzyme E2N plays a crucial part in cell cycle regulation, progression, inflammation, error-free DNA repair, differentiation, and metastasis (24, 25). Recent studies demonstrate the involvement of UBE2N in progressive cases of melanoma, HCC, breast, prostate, lymphoma, and ovarian cancer. Similar to our hypothesis, reports have shown UBE2N is essential for breast cancer metastasis to the lungs *in vivo* through TGF-β mediated activation of Tak 1 and p38. Another study has reported that UBE2N promotes melanoma growth via MEK/FRA1/SOX10 signaling hence proving the role of UBE2N as a potential biomarker for PCa (25). Microarray studies have shown upregulated expression of UBE2N in TRAMP mice when compared to their age-matched non-transgenic littermate (26). Another group has also reported the involvement of UBE2N at protein level in rat spermatogenesis in response to *in vivo* androgen manipulation (27).

Among the identified proteins, p21-activated kinase 2 (PAK2) is known to be a member of the PAK family of Serine/Threonine kinase localized in both cytoplasmic and nuclear compartments. PAK2 has been known to play critical roles in many fundamental functions including chromatin remodeling, proliferation, and regulation of cellular apoptosis by mediating proteolytic cleavage during cancer mediated apoptosis (28). Studies have shown elevated expression of PAK2 resulting in an alteration of histone modification, thereby regulating gene expressions also. Differential expression levels of PAK2 have been reported in various malignancies including breast, gastric, hepatocarcinoma and head, and neck cancer (28, 29). Elevated levels of PAK2 have been reported in castration-resistant tumors and the knockdown experiments further revealed that PAK2 can regulate colony formation and invasion experiment. Further pharmacological inhibitors of PAK2 viz., PF-3758309 could be seen to inhibit the growth of androgen independent PC-3 xenografts (30). Hence, PAK2 could be studied further as a therapeutic target for reducing cellular proliferation and acquired chemo-resistance (30). The survival analysis further showed that high expression levels of PAK2 led to a reduction in the long-term survival of PCa patients (Figure 5).

The mitogen-activated protein kinase 1 is a crucial part of the MAP kinase signaling pathway. The MAPK cascade is known to play an integral role in regulating diverse biological and MF viz., cell growth, differentiation, adhesion, survival, apoptosis, and translation (31). MAPK signaling is known to be among the most differentially regulated signaling pathways in various cancers including cervical cancer, lymphoma, prostate, head, and neck cancers. Evidences have shown MAPK1 signaling promotes EMT as well (32). Another report showed that PD0325901, an inhibitor of MAPK kinase 1 in combination with rapamycin could significantly inhibit tumor growth in androgen independent prostate tumors in mouse models (33). The role of MAPK1 in hepatocyte proliferation has been reported both in *in vitro* and *in vivo* models (34). Based on our analysis, MAPK1 was seen to be involved in regulation of actin cytoskeleton, focal adhesion, adherens junction, dorsoventral axis formation, mitotic M-M/G1 phase cell cycle, intracellular protein transport, and establishment of localization in cells thereby pointing toward the involvement of MAPK1 with the progression of PCa toward metastasis.

The heterogeneous nuclear riboprotein A2/B1 is an oncogene that controls the sorting of miRNAs into exosomes through binding to specific motifs. Evidence has suggested HNRNPA2B1 playing a direct role in cancer initiation, development, gene expression, and signal transduction (35). HNRNPA2B1 affects the major hallmarks of cancer by promoting proliferative signaling, change of cellular energetics, and suppressing tumor-promoting inflammation and invasion and metastasis. HNRNPA2B1 contributes in activating cyclo-oxygenase 2, which eventually leads to tumor growth, promoting EMT through ERK/SNAIL signaling reduced cell proliferation and prolonged S-phase and suppressed subcutaneous tumorigenicity. Knockdown of HNRNPA2B1 can lead to suppression in subcutaneous tumors *in vivo* models (35). Another study revealed that HNRNPA2B1 promotes EMT by downregualting E-cadherin and upregulated of mesenchymal markers such as N-cadherin and vimentin and also promotes invasion potential at *in vitro* and *in vivo* BALB/C-nu/nu mice (36). Differential expression of HNRNPA2B1 has been reported in breast cancer, PCa, pancreatic cancer, and non-small cell lung cancer (36). Our analysis further supported the potential of PAK2 as a biomarker for PCa since the high expression of HNRNPA2B1 was seen to be significantly reducing the survival of PCa patients (Figure 5).

Another identified protein in our study, YWHAB encodes for a protein belonging to the 14-3-3 protein family, which mediates signal transduction by binding to proteins containing phosphoserine (37). The encoded protein interacts with Raf1 and Cdc25 phosphatases indicating toward its association with cell cycle machinery and mitogenic signaling. The gene enrichment analysis showed YWHAB to be involved with cadherin binding, enzyme binding, histone deacetylase binding, Hippo signaling, and MAPK cascade. Differential expression of YWHAB has been reported in ovarian cancer, lung cancer, breast, and PCa (38).

Glutamate oxaloacetate transaminase (I) (GOT1), an important regulator of glutamate levels, is involved in the biosynthesis of L-glutamate from L-aspartate, or L-cysteine (39). GOT 1 has also been reported playing a role in energy metabolism and ROS balance in chronic acidosis stress. Differential expression levels of GOT1 have been reported in several cancers including breast (40), lungs, brain, and colorectal cancer. Similar to our gene enrichment analysis, other meta-analysis studies have shown significant roles of GOT1 in arginine and proline metabolism and also alanine, aspartate and glutamate metabolism, and TCA cycle in three PCa cell lines datasets. Knockdown of GOT1 has been known to suppress tumor growth, invasiveness, colony-forming ability, and also cell viability of PC-3 (androgen independent) and LNCaP (androgen independent) cells. Elevated levels of GOT1 have also been observed in malignant high Gleason score Prostate tumors when compared to controls. Thereby implying that PCa cells may be undergoing GOT1 dependent metabolism while acquiring malignant phenotype and hence pointing toward GOT1 as a potential biomarker for PCa (41). GOT1 was reported to be downregulated (Average fold −1.5) in hormonal treated androgen independent CaP xenografts (LuCaP 35V) when compared to their untreated xenografts by cDNA microarray analysis, further pointing toward the involvement of GOT1 in immune response and androgen receptor signaling (42). Another study has demonstrated the importance of GOT1 in tumor growth *in vivo* tumor models of CRC (43).

It should be noted that complex metabolic programming in PCa revealed by proteomic data analysis showed dysregulation of various crucial pathways including TCA cycle, pentose phosphate pathway, Fructose and mannose metabolism which are known to contribute in tumor initiation and progression and hence shedding light on a distinct metabolic exhibit of PCa. Furthermore, MF such as cell cycle, apoptosis, DNA integrity checkpoint, cell adhesion, cadherin binding, and actin-binding which are known to affect the transition of androgen dependent to androgen-independent stages were seen to be dysregulated by our identified DEPs, hence depicting the involvement and contribution of protein markers in tumorigenesis and further vouching for their potential as biomarkers for PCa prognosis and diagnosis. The high-level expressions of the identified DEPs were seen to reduce the long-term survival of PCa patients.

## Conclusion

In conclusion, we compared the proteomic profiles of the effect of induced EMT post-TGF-β treatment in androgen dependent and androgen independent PCa using a SWATH-LC-MS/MS quantification strategy. To obtain results, protein lysate samples were subjected to SWATH analysis under strict filtration criteria. In total, 1795 among which 474 proteins were seen to be significantly deregulated in both cases and were subsequently subjected to Gene ontology and network analysis. The shortlisted identified DEPs were then reviewed under differential expression and survival analysis to evaluate their contribution in long-term survival of PCa, which led to the identification of six proteins as potential therapeutic and diagnostic biomarkers for PCa.

In total, six crucial hub proteins identified in our study viz., GOT1, HNRNPA2B1, MAPK1, PAK2, UBE2N, and YWHAB seen to be significantly contributing in cancer development and progression from an androgen dependent to androgen independent stage, and were found to affect overall long-term survival of patients. The differential expression of these proteins in PCa tissue samples further supported the role of these proteins as potential biomarkers. These DEPs enriched in the hallmarks of cancer can act as potential diagnostic and prognostic biomarkers in cancer therapy and thereby can contribute to therapeutic drug-target discovery.

## Data Availability Statement

The raw data supporting the conclusions of this article will be made available by the authors, without undue reservation, to any qualified researcher.

## Author Contributions

AS and NS: conceptualization, methodology, validation, formal analysis, investigation, data curation, writing-original draft preparation, and writing-review and editing. NS: resources, supervision, project administration, and funding acquisition. All authors approved the final manuscript.

### Conflict of Interest

The authors declare that the research was conducted in the absence of any commercial or financial relationships that could be construed as a potential conflict of interest.
