# Supplementary material for: Quantitative SWATH-Based Proteomic Profiling for Identification of Mechanism-Driven Diagnostic Biomarkers Conferring in the Progression of Metastatic Prostate Cancer
Source: Front Oncol. 2020 Apr 8;10:493. doi: 10.3389/fonc.2020.00493 (PMC7156536; doi:10.3389/fonc.2020.00493)

Supplementary Figures

Figure S1: MTT assay results for treatment of PC-3 and LNCaP cells with TGF-β for 24 hours (All the experiments were done in triplicates) *P*-value at <0.05 was considered to be statistically significant.

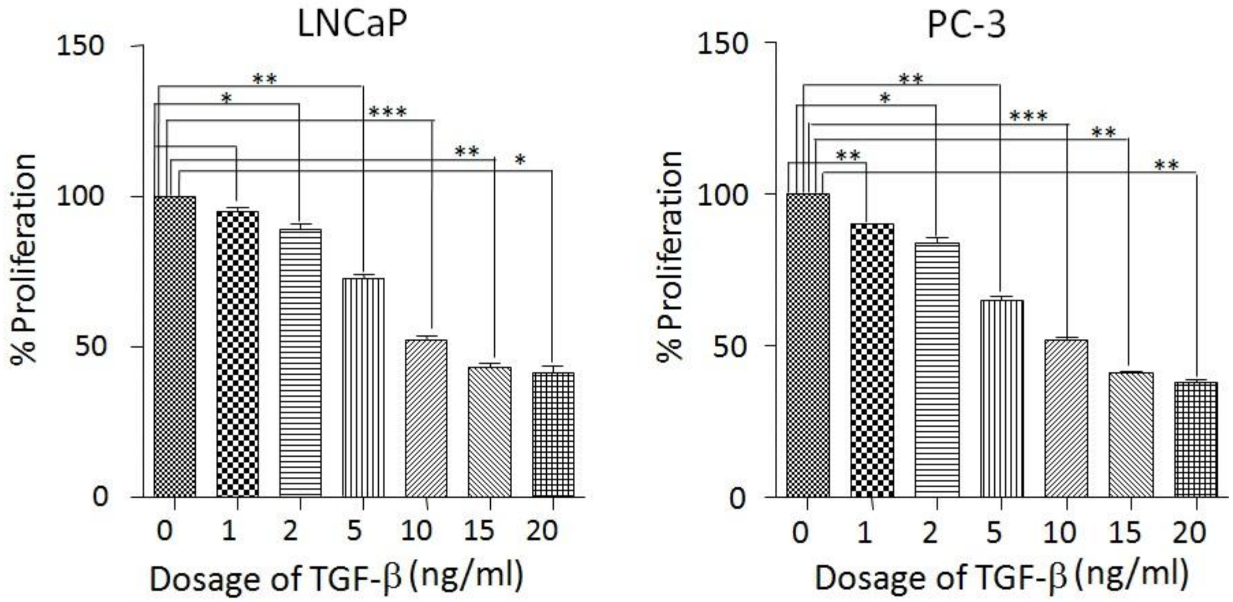

Figure S2: TGF-β downregulates epithelial marker expression (E-cadherin) and promotes mesenchymal marker expressions (N-cadherin and Vimentin). Quantitative RT-PCR analysis showing mRNA expressions of EMT markers in (a) androgen dependent LNCaP and (b) androgen independent PC-3 Prostate cancer cell lines in naïve and post TGF-β treatment conditions. The real-time PCR data are normalized to the β-actin reference gene and were analyzed by the comparative CT method. *P*-value at <0.05 was considered to be statistically significant. (c) Western blot data showing the expression of EMT markers (E-cadherin and N-cadherin) in LNCaP and PC-3 Prostate cancer cell lines in naïve and post TGF-β treatment conditions using β-actin as internal control.

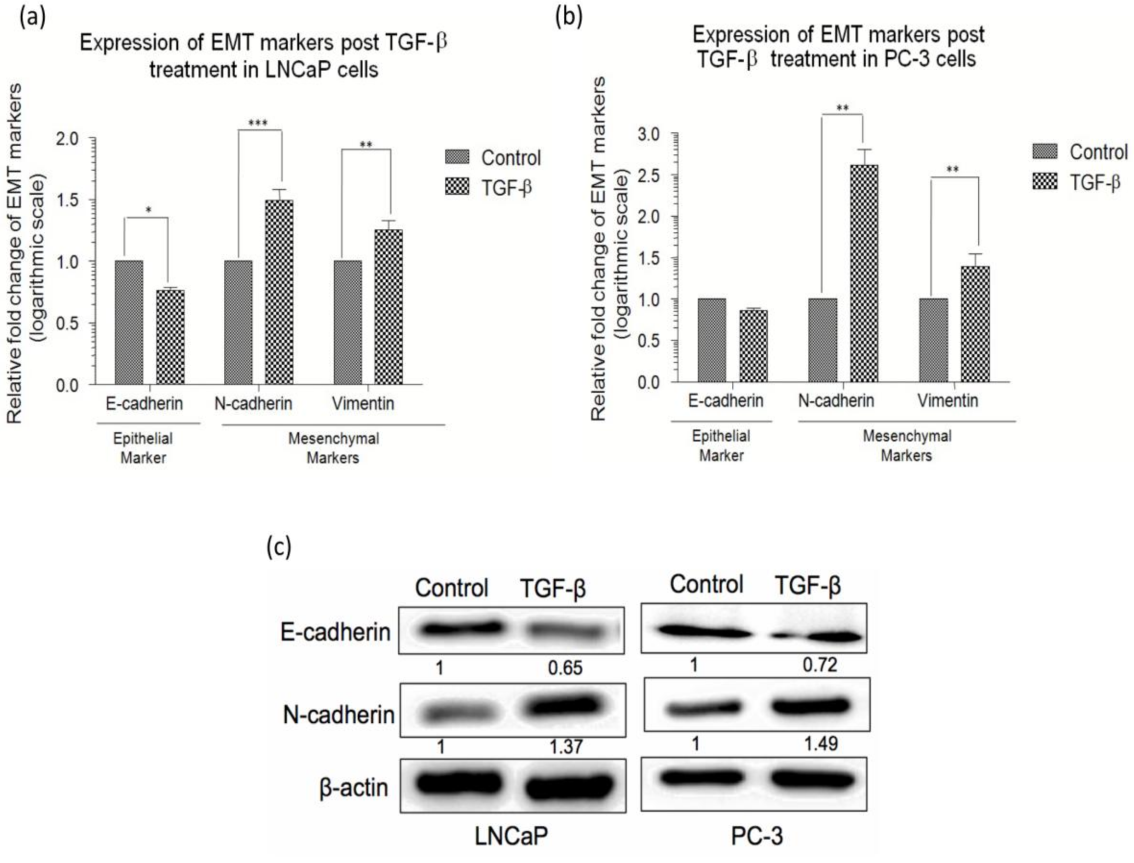

Figure S3: Network correlation of most deregulated pathways in Androgen dependent LNCaP cell line (The Purple and Pink dots represent the co-modulated functions by identified proteins and the grey lines represent the connection between the functions).

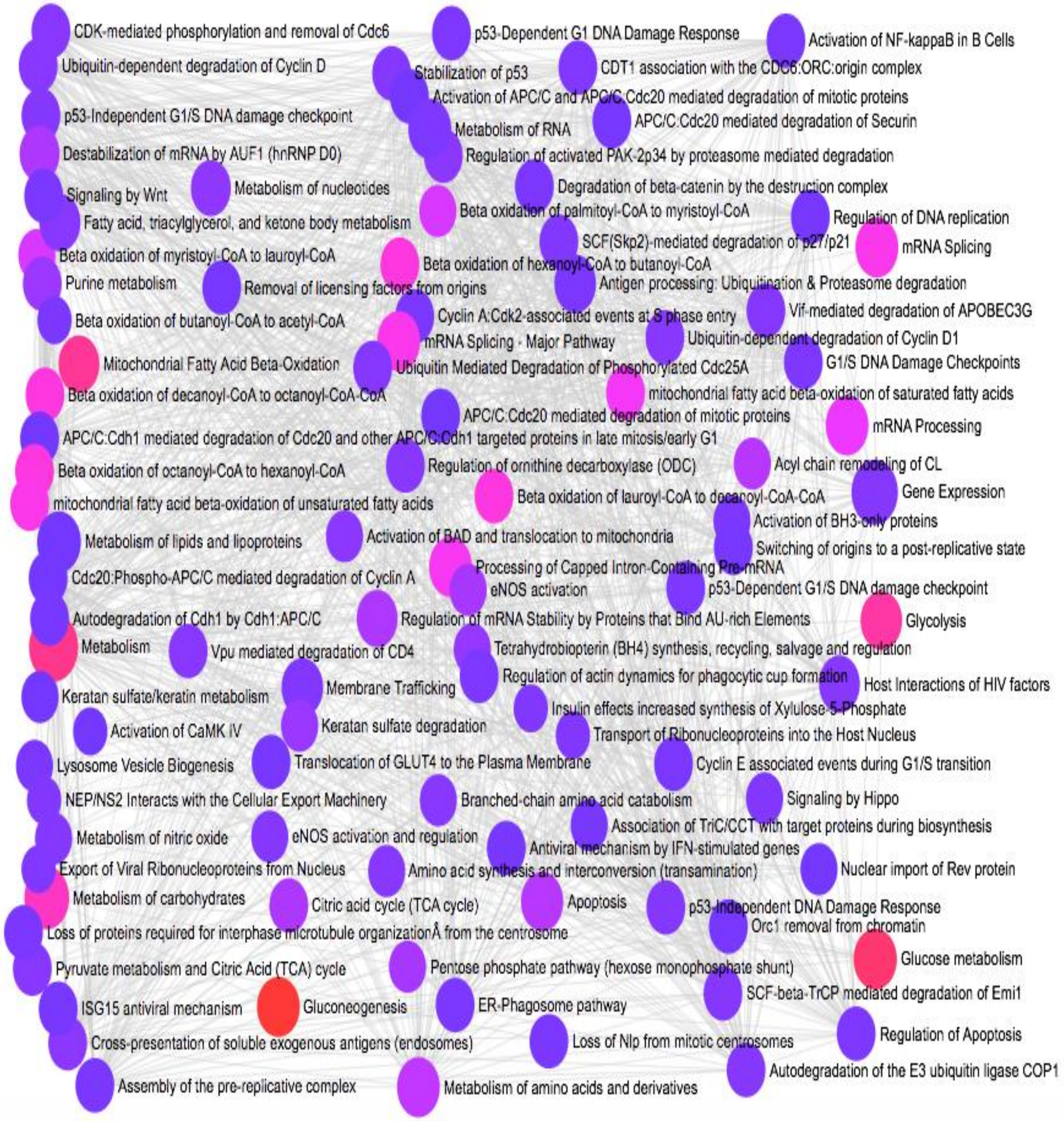

Figure S4: Network correlation of most deregulated pathways in Androgen independent PC-3 cell line (The Purple and Pink dots represent the co-modulated functions by identified proteins and the grey lines represent the connection between the functions).

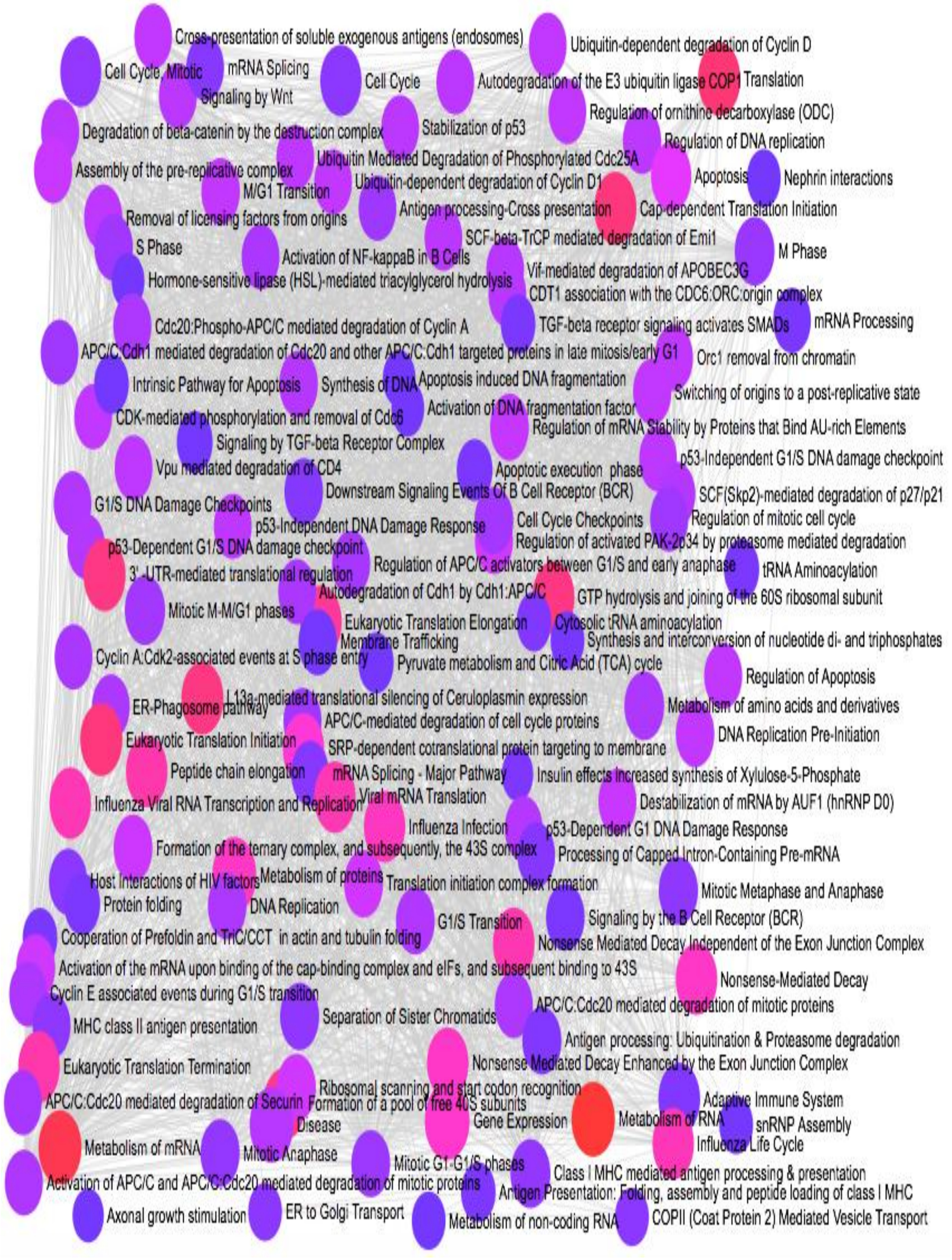

Figure S5: Protein–protein interactions (PPI) of the differentially expressed proteins in prostate cancer. Colored lines indicate the type of inter- action evidence between nodes

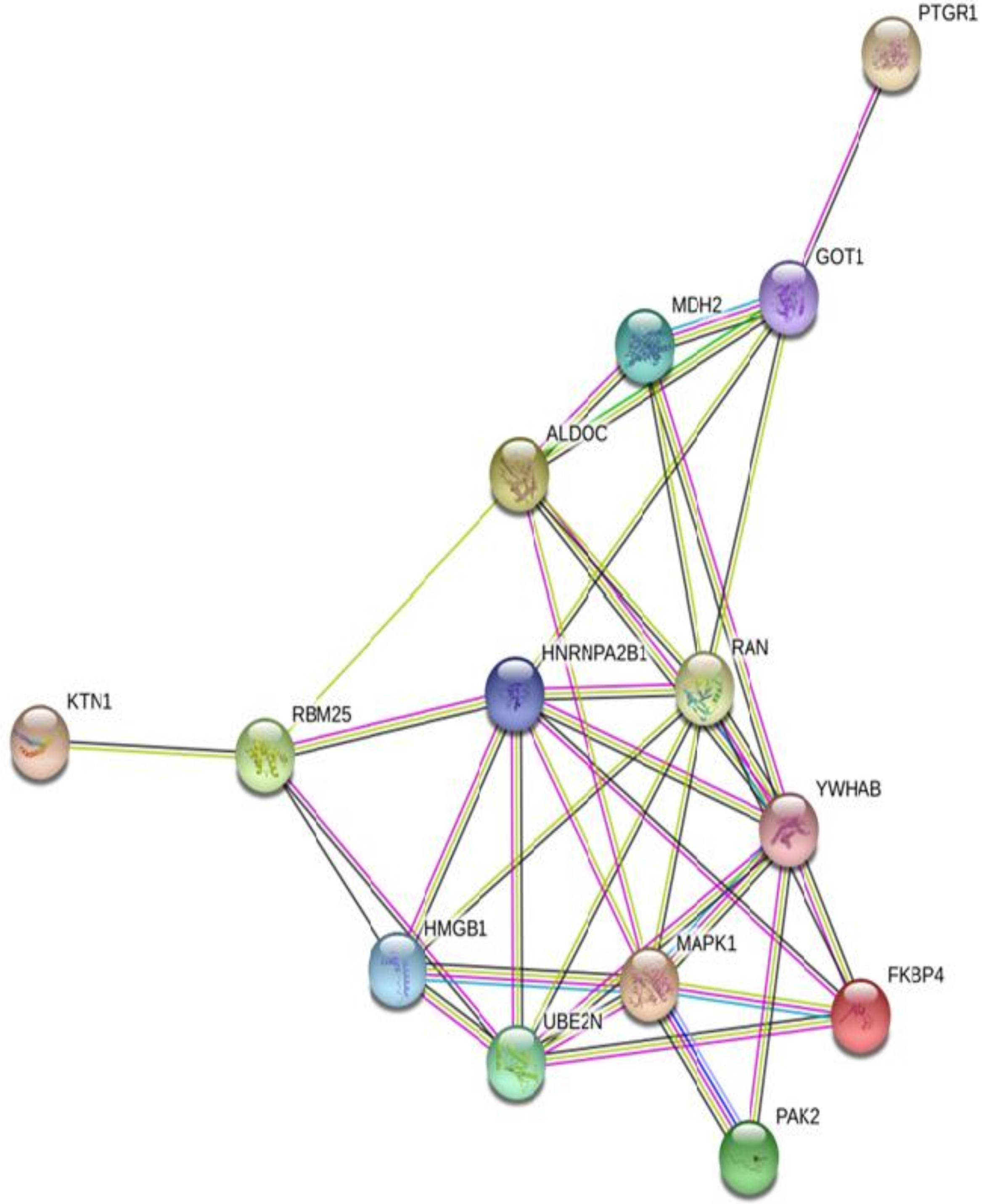

Supplement: Supplementary file 1 [file Data_Sheet_1.pdf]
